# The quantification of *COMT *mRNA in *post mortem *cerebellum tissue: diagnosis, genotype, methylation and expression

**DOI:** 10.1186/1471-2350-7-10

**Published:** 2006-02-16

**Authors:** Emma L Dempster, Jonathan Mill, Ian W Craig, David A Collier

**Affiliations:** 1 Molecular Genetics, Psychological Medicine, Institute of Psychiatry, London, UK; 2 SGDP Research Centre, Institute of Psychiatry, London, UK

## Abstract

**Background:**

The *COMT *gene is located on chromosome 22q11, a region strongly implicated in the aetiology of several psychiatric disorders, in particular schizophrenia. Previous research has suggested that activity and expression of *COMT *is altered in schizophrenia, and is mediated by one or more polymorphisms within the gene, including the functional Val^158^Met polymorphism.

**Method:**

In this study we examined the expression levels of *COMT *mRNA using quantitative RT-PCR in 60 *post mortem *cerebellum samples derived from individuals with schizophrenia, bipolar disorder, depression, and no history of psychopathology. Furthermore, we have examined the methylation status of two CpG sites in the promoter region of the gene.

**Results:**

We found no evidence of altered *COMT *expression or methylation in any of the psychiatric diagnoses examined. We did, however, find evidence to suggest that genotype is related to *COMT *gene expression, replicating the findings of two previous studies. Specifically, val^158^met (rs165688; Val allele) rs737865 (G allele) and rs165599 (G allele) all showed reduced expression (P < 0.05). Finally, we observe a strong sexual dimorphism in *COMT *expression, with females exhibiting significantly greater levels of *COMT *mRNA.

**Conclusion:**

The expression of *COMT *does not appear to be altered in the cerebellum of individuals suffering from schizophrenia, bipolar disorder or depression, but does appear to be influenced by single nucleotide polymorphisms within the gene.

## Background

There has been considerable progress in the search for genes involved in the aetiology of psychiatric disorders such as schizophrenia. Whilst associations have been made between polymorphic variants in various genes (e.g. Neuregulin, Dysbindin, *COMT*), it is generally not clear whether the associated polymorphisms are the precise functional variants mediating susceptibility [1]. In this study we have examined expression of the Catechol-O-Methyltransferase (*COMT*) gene, primarily associated with schizophrenia, but also linked to other forms of psychopathology and variation in normal prefrontal cognitive functioning (see [1-4]). COMT is a widely expressed enzyme that metabolises released dopamine in the synapse. In the prefrontal cortex it is the main component in the dopamine metabolic pathway, since the dopamine transporter is not abundant in this brain region [5-7]. There are two known promoters in *COMT *that encode two different mRNAs, a longer membrane-bound *COMT *(MB-COMT) and a shorter soluble *COMT *(S-COMT) [8].

Although *COMT*, which maps to the velo-cardio-facial syndrome region located at 22q11, has a well characterised functional mutation that alters enzyme activity (val^158^met) and is responsible for the majority of variation in serum COMT activity levels, it is unclear if this mutation is solely responsible for the association of this gene and schizophrenia. The met^158 ^allele has been shown to produce an enzyme with reduced thermostability [9,10], exhibiting about 60% of the activity of the val^158 ^allele in the brain at normal body temperature [11]. The alleles are co-dominant, with heterozygotes exhibiting intermediate activity [12].

Li et al. [13] first found association between schizophrenia and the val^158 ^allele, and although there have been some replications [1,14-16] association results with schizophrenia have been inconsistent, and largely negative for depression and bipolar disorder. A recent meta-analysis of the common val^158^met polymorphism and schizophrenia failed to find overall evidence for a significant association [17,18]. Recent focus has thus shifted to haplotype analysis of the *COMT *gene in schizophrenia. Li et al. [19] examined an extended set of markers in the gene and found strong haplotypic association, which is supported by subsequent haplotype studies by others [20-22]. Population analysis of the previously typed variants showed widely differing allele and haplotype frequencies between populations. It has been postulated that variation in linkage disequilibrium may account for the inconsistencies seen in previous association studies focussing on the val^158^met SNP [23]. It also indicates that either the val^158^met polymorphism may be a surrogate for a further functional variant involved in the aetiology of schizophrenia, or there are two sites of functional variation in the gene which are relevant to psychosis [19]. Shifman et al. (2002), while finding modest evidence of an association with the val^158^met SNP, identified significant association between schizophrenia and a *COMT *haplotype consisting of three SNPs, including val^158^met, in a large case-control population consisting of Ashkenazi Jews [21]. Functional studies on *COMT *performed by Bray et al. found that this haplotype is associated with a decrease in *COMT *mRNA expression [24], an observation that has been replicated by some groups [25], but not by others (e.g. [11,26]). Handoko et al. (2005) also found haplotypic association with schizophrenia, with evidence for separate and interacting effects of SNPs in COMT on susceptibility to the disease [20]. The success of haplotype analysis of the gene along with expression work performed by Bray et al. suggests that *COMT *regulatory regions may be important in schizophrenia, warranting further expression studies of the gene.

It has been recently suggested that epigenetic factors may a key role in the development of schizophrenia [27], and these processes may explain the inconsistent association-study results often observed for variants in the *COMT *gene. Of particular interest is the phenomenon of cytosine methylation at CpG sites, a molecular process that is intrinsically linked to the regulation of gene expression. Methylation at CpG sites, principally located in CpG-islands in the promoter regulatory regions of many genes, disrupts the binding of transcription factors and attracts methyl-binding proteins that are associated with gene silencing and chromatin compaction. Murphy et al. have recently examined the methylation status of six CpG sites in the promoter region of *COMT *[28]. Using bisulfite-genomic sequencing they found that four of the sites were totally methylated in all individuals assessed, but that two adjacent CpG sites (incorporating cytosines 23 and 27 in their analysis) showed evidence for only partial methylation and some degree of between-individual variation.

In this study we have investigated the role of *COMT *expression in the development of schizophrenia and two aetiologically-related psychiatric disorders: bipolar affective disorder and depression. We have investigated the relative expression level of *COMT *in cerebellum tissue obtained from *post mortem *psychiatric patients. Furthermore, we have correlated gene expression levels with genotype for each of the major candidate susceptibility polymorphisms. Finally, we have accurately quantified the degree of methylation at the two *COMT *promoter CpG sites to see if epigenetic factors affect gene expression levels and susceptibility to psychopathology.

## Methods

### Samples

We obtained 60 *post mortem *cerebellum brain samples from The Stanley Medical Research Institute's brain collection [29]. 15 samples had a diagnosis of schizophrenia, 15 bipolar, 15 depression and 15 controls had no history of mental illness.

### Sample preparation

Both DNA and mRNA were extracted simultaneously using Trizol following the manufacturer's standard protocol (Invitrogen, UK). To ensure no DNA contamination, clean-up of the RNA was performed using QIAGEN spin columns with an additional DNAase step (QIAGEN, Crawley, UK). The quality and purity of total RNA was assayed in a 2% agarose gel and the recovery was calculated after measuring absorbance with a spectrophotometer at 260 nm. Reverse Transcription was performed using TaqMan reverse transcription reagents with random hexamers (Applied Biosystems, Foster City, USA). All cDNA samples were tested for genomic DNA contamination by PCR amplification of a non-transcribed sequence. Samples were stored at -70°C prior to further use.

### Quantitative PCR

Quantitative RT-PCR was performed in triplicate for each sample on an ABI Prism 7900HT with TaqMan universal PCR master mix (Applied Biosystems, Foster City, USA) using a standard protocol. An Assay-By-Demand probe/primer set specific to *COMT *(Hs00241349 _m1), and a β-Actin housekeeping control gene probe were obtained from Applied Biosystems (Applied Biosystems, Foster City, USA). The expression data produced were analysed and converted into threshold cycle values (Ct-values) using SDS 2.0 (Applied Biosystems, Foster City, USA). The comparative C_T _method (ΔΔC_T_) was used to measure the relative gene expression. In this method mathematic formulas are used to calculate relative expression levels in comparison to a 'calibrator' (e.g. a different diagnosis group). An arbitrary fluorescence threshold is chosen, based on the variability of the background, and threshold cycle (C_T_) values are calculated by determining the cycle number at which the fluorescence exceeds this threshold. C_T _values decrease with increasing input target quantity, thus, providing a quantitative measure. The amount of target, normalised to an endogenous housekeeping gene and relative to a calibrator is then given by the formula 2^-ΔΔCT^.

### CpG methylation analysis

Cerebellum DNA was treated with sodium bisulfite using a modified version of the protocol outlined by Olek et al. [30]. Briefly, ~800 ng samples of genomic DNA (in a volume of 21 μl) were denatured at 95°C for 10 minutes, followed by incubation with 4 μl 2 M NaOH solution at 50°C for 15 minutes. DNA was mixed with 50 μl of 2% low-melting agarose, and 8 μl beads were formed in pre-chilled mineral oil. Bisulfite conversion was performed with a 5 M sodium bisulfite solution at 50°C for 4 hours, under exclusion of light. The beads were washed twice for 15 minutes in TE buffer (pH = 8.0). Desulfonation was done in 0.2 M NaOH, twice for 15 minutes each, followed by two additional washing steps, again, with TE buffer. Single beads were washed with water and used for subsequent PCR reactions. Two primers, specific to bisulfite-treated DNA, were designed on the reverse-complement strand to flank the two *COMT *CpG sites (F: 5'-GAG TAG GTT GTG GAT GGG TTG TA-3' and R: 5'-*biotin*-ACA TTT CTA AAC CTT ACC CCT CTA-3'). Purification with streptavidin beads and Pyrosequencing™ (Biotage, Uppsala, Sweden) reactions were performed according to the manufacturer's standard protocol using the sequencing primer 5'-GTA ATA TAG TTG TTA ATA GTA GA-3'.

### Genotyping

Three common *COMT *SNPs (val^158^met/rs165688, rs737865, and rs165599) were genotyped using the Amersham SNuPe primer extension assay (GE Healthcare, UK), and products separated on a MegaBACE capillary system (GE Healthcare, UK). Primers and assay conditions are available on request from the authors.

### Statistical analysis

Spearman's rank correlations were calculated for the relationship between *COMT *expression and all available demographic data to determine if any other factors were contributing to the variation seen in expression. Significant correlations were found with the variables: gender (Rho = -0.366 P = 0.005) and age at death (Rho = -0.340 P = 0.01). To ensure the variation in gender, age and genotype was not confounding the expression differences seen for the different SNP genotypes, ANCOVA was performed with age, gender and genotype as covariates where appropriate. All statistical analyses between groups were performed using SPSS (version 10.0).

## Results

Table 1 describes the expression data observed for *COMT*. Table 2 describes the association findings. No association was found between disease status and *COMT *expression (F = 0.412, 3 df, p = 0.745). However, genotype at all three *COMT *SNPs was found to be associated with gene expression (p < 0.05), with the G allele of each polymorphism being correlated with decreased expression. Haplotype analysis gave further evidence for an association between *COMT *genotype and expression with the G-G-G haplotype being significantly correlated with reduced expression (F = 4.25, 1 df, p = 0.04). We found some evidence to suggest that *COMT *is expressed at higher levels in females than males (F = 5.97, 1 df, p = 0.02). Average CpG methylation status across all brain samples were 45.4% for site 1 (cytosine 27 in the study of Murphy et al. [28]) and 34.5% for site 2 (cytosine 23). Methylation levels at the two sites were highly correlated (r = 0.8, p =< 0.000). Quantitative measurement of the methylation status of the two promoter CpG sites suggested that there was no association between methylation level and either disease status, or expression level. We did, however, find some preliminary evidence that methylation level was associated with val^158^met at site 1 (Regression coefficient 0.26; t= 2.08; R^2 ^= 0.072; *P *= 0.042), the relationship was dose-dependent with val^158 ^homozygotes exhibiting lower levels of methlyation at site 1 than met^158 ^homozygotes. The same relationship was observed at the second CpG site, although not significant. (Regression coefficient 0.19; t= 1.4; R^2 ^= 0.021; *P *= 0.1).

**Table 1:** Relative expression for gender and the three SNPs analysed using the comparative C_T _method (ΔΔC_T_).

**Variable**	**Control Group (Relative expression = 1)**	**Other group (Relative expression compared to the control group, ± SE)**	
Gender	Female	Male	0.77 (± 0.07)
val^108/158^met genotype	val/val	val/met	1.5 (± 0.11)
		met/met	1.3 (± 0.09)
SNP rs_737865 genotype	AA	AG/GG	0.7 (± 0.07)
SNP rs_165599 genotype	AA	AG/GG	0.8 (± 0.07)
COMT haplotype (GGG)	Haplotype absent	Putative haplotype carrier	0.7 (± 0.06)

**Table 2:** ANCOVA analysis of *COMT *gene expression

**Variable**	**F**	**df**	**Significance P value**
val^158^met (rs165688) (val/val versus val/met and met/met combined)	5.7	1	0.020
rs737865 (AA versus AG and GG)	5.134	1	0.028
rs165599 (AA versus AG and GG)	4.18	1	0.046
*COMT *haplotype (GGG)* *Shifman et al. (2002) "at risk haplotype"	4.25	1	0.044
Gender	5.97	1	0.018
Diagnosis	0.412	3	0.745

## Discussion

In this study we have examined the expression of *COMT *in *post mortem *cerebellum tissue from individuals diagnosed with schizophrenia, bipolar disorder, and depression. Furthermore, we have examined the role of three polymorphic variants and methylation status at two promoter CpG sites in mediating levels of *COMT *gene expression. We found no difference in *COMT *expression or methylation levels in any of the diagnostic groups. However, we found some evidence to suggest that genotype at the three SNP polymorphisms is correlated with expression level in the cerebellum.

We found evidence that the SNPs constituting the Shifman et al. "at risk" schizophrenia haplotype [22] are associated with changes in *COMT *mRNA expression. This finding is in agreement with those reported by Bray et al. whose data suggest that the specific *COMT *haplotype implicated in schizophrenia is associated with lower expression of *COMT *mRNA [24], a finding also replicated by Zhu et al. [25]. Our study is the first positive report that has compared individual genotypes with actual expression levels, as these previous investigations [24,25] both examined relative allele-specific expression in heterozygous individuals. It should be noted, however, that there are two non-replications of the original Bray et al. finding. Tunbridge et al. examined mRNA derived from the dorsolateral prefrontal cortex [26]. Using Northern blotting, they were unable to distinguish any differences in expression levels between diagnostic groups, or in the genotypic groups of the val^158^met SNP. Chen et al. examined *COMT *expression levels in *post mortem *prefrontal cortex tissue using real-time quantitative PCR, and also failed to identify any association between any *COMT *polymorphism and mRNA expression [11]. They did, however, find that the val^158^met polymorphism significantly affected protein abundance and enzyme activity independently of mRNA expression, confirming the thermolability of the met substitution [11].

A number of factors could explain these inconsistent data. Our study is the only one to examine gene expression in the cerebellum, and it is possible that tissue-specific gene-expression differences exist. Furthermore, gene association studies of *COMT *in schizophrenia do not ubiquitously support an aetiological role for variants within the gene. It is possible that another functional variant elsewhere in the gene is mediating the putative val^158^met association in some populations via linkage disequilibrium (LD). The observation that *COMT *variants, in particular the val^158^met SNP, are in strong LD with variants in the nearby *ARVCF *gene suggest that the alterations in expression associated with particular *COMT *genotypes seen our data and previous studies by Bray et al. [24] and Zhu et al. [25] may be mediated by variants not yet fully investigated. Further work should examine variants in the VCFS region, in particular the "at risk haplotype" implicated by Sanders et al. that spans the 3'region of *COMT *and *ARVCF *[21], to determine if any of these variants could be *cis *acting factors influencing altered *COMT *expression, or to substantiate *ARVCF *as a candidate gene in its own right. Overall, given that the met^158 ^allele encodes an enzyme with reduced thermostability, exhibiting about 60% of the activity of the val^158 ^allele [11], and that the observed mRNA expression levels associated with this SNP are not nearly so pronounced, it is likely that this polymorphism does not have a direct effect on transcription through for example altering mRNA stability or transcriptional efficiency.

Interestingly, no association has been identified between *COMT *expression and disease status in any recent study so far. It has been postulated that schizophrenics may show a decrease in *COMT *expression [31], paralleling the 22q11 haploinsufficiency seen in individuals with VCFS, who are at greater risk of developing schizophrenia. Overall, the available data suggest there is a haplotype spanning *COMT *associated with a slightly elevated risk to schizophrenia, which confers a decrease in expression in *COMT *mRNA. Because of the absence of any noticeable decrease in the schizophrenia diagnostic group, it is probable that this decrease in *COMT *is only present in a subsection of the schizophrenia patients, and presents only a small but detectable increase in risk of developing the disorder. However the recent finding of a gene-environment effect between *COMT *val^158^met and cannabis use in schizophrenia may help to clarify these association findings [32].

It has been widely postulated that conflicting findings in genetic studies of schizophrenia and other complex diseases may be a result of epigenetic factors [33]. In this regard, we decided to investigate the methylation status of two CpG sites in the S promoter region of the *COMT *gene, first identified by Murphy et al [28]. CpG methylation is a molecular process that is intrinsically linked to the regulation of gene expression. Methylation at CpG sites, principally located in CpG-islands in the promoter regulatory regions of many genes, disrupts the binding of transcription factors and attracts methyl-binding proteins that are associated with gene silencing and chromatin compaction. Petronis et al. have argued that these epigenetic mechanisms can explain a number of the non-Mendelian features observed in a range of complex psychiatric disorders such as schizophrenia [33]. Unlike DNA sequence variation, which is generally highly stable, epigenetic processes are highly dynamic: they can be tissue-specific, developmentally-regulated, and induced by exposure to a range of environmental stressors. We found that there was no difference in the methylation status of these two CpG sites in any of the diagnostic groups examined. Furthermore, we found no evidence to suggest that methylation of either of these two sites was strongly correlated with *COMT *expression levels. Interestingly, we did observe a correlation between *COMT *genotype and methylation levels. Whilst this finding is likely to be a spurious artefact, it is possible that this reflects a difference between the *COMT *methylation status of each individuals' two chromosomes, potentially indicative of some form of imprinting.

In this regard it is interesting that in contrast to previous studies, we found a significant effect of gender on expression levels, with females exhibiting higher expression of *COMT *than males. Reasons for this inconsistency between studies could be explained by smaller sample size and the difference in brain region studied. Tunbridge et al. [26] along with Bray et al. [24] both investigated mRNA extracted from prefrontal cortex tissue, raising the possibility that the gender effect is only evident in the cerebellum. There is substantial evidence supporting sexual dimorphism in *COMT*. Most notably, the G-G-G haplotype association identified by Shifman et al. was principally driven by females [22]. Gogos et al. demonstrated a sexual dimorphism between homozygous *COMT *deficient mice, with females exhibiting behavioural deficits in anxiety tasks in comparison to males [34], while heterozygous COMT deficient males exhibited increased aggressive behaviour. Furthermore, Karayiorgou et al. found a sexual dimorphic pattern of allele transmission in Obsessive-Compulsive Disorder (OCD) and *COMT *val^158^met polymorphism: males showed a significant association with the met allele, where females showed no association with either allele [35,36]. COMT is involved in the metabolism of catechol-oestrogens [37], which may explain why we observe that the expression level of *COMT *is elevated in females. The sexual dimorphism of *COMT *expression, and the possibility of genomic imprinting, is an area that needs further research before any firm conclusions can be made.

The main limitation of this work is the lack of specificity of the *COMT *probe used to any one form of *COMT*. MB-COMT is predominantly the form found in brain [38] but how these forms differ in different brain regions to what degree is unknown. Further work should investigate these findings relevant to the two different *COMT *species.

## Conclusion

To conclude, we have examined the role of *COMT *expression in the aetiology of schizophrenia, depression, and bipolar disorder. We find no evidence to suggest that *COMT *expression is altered in any of these forms of psychopathology. Furthermore, we find no difference in *COMT *promoter methylation patterns in any diagnostic category, and find no evidence to suggest that CpG methylation is directly correlated to gene expression. However, we do find evidence to suggest sexual dimorphism in the expression of the *COMT *gene, and replicate previous reports implicating polymorphisms in the *COMT *gene in the regulation of mRNA expression.

## Competing interests

The author(s) declare that they have no competing interests.

## Authors' contributions

ED performed the majority of the lab-work, data-analysis and drafted the manuscript. JM helped with the expression and CpG methylation analysis and helped produce the final manuscript. DC and IC participated in the overall design and co-ordination of the study and contributed to the interpretation of findings. All authors read and approved the final manuscript.

## Pre-publication history

The pre-publication history for this paper can be accessed here:


